# Venous vascular closure system vs. figure-of-eight suture following atrial fibrillation ablation: the STYLE-AF Study

**DOI:** 10.1093/europace/euae105

**Published:** 2024-04-22

**Authors:** Roland Richard Tilz, Marcel Feher, Julia Vogler, Kerstin Bode, Alexandru Ionut Duta, Angela Ortolan, Lisbeth Delgado Lopez, Mirco Küchler, Roman Mamaev, Evgeny Lyan, Philipp Sommer, Martin Braun, Vanessa Sciacca, Thomas Demming, Vera Maslova, Karl-Heinz Kuck, Christian-Hendrik Heeger, Charlotte Eitel, Sorin Stefan Popescu

**Affiliations:** Department of Rhythmology, University Heart Center Lübeck, University Clinic Schleswig-Holstein, Ratzeburger Allee 160, D-23538 Luebeck, Germany; German Center for Cardiovascular Research (DZHK), Partner Site Hamburg/Kiel/Lübeck, Luebeck, Germany; Department of Rhythmology, University Heart Center Lübeck, University Clinic Schleswig-Holstein, Ratzeburger Allee 160, D-23538 Luebeck, Germany; Department of Rhythmology, University Heart Center Lübeck, University Clinic Schleswig-Holstein, Ratzeburger Allee 160, D-23538 Luebeck, Germany; Department of Rhythmology, Heart Center of Leipzig, Leipzig, Germany; Department of Rhythmology, University Heart Center Lübeck, University Clinic Schleswig-Holstein, Ratzeburger Allee 160, D-23538 Luebeck, Germany; Department of Rhythmology, University Heart Center Lübeck, University Clinic Schleswig-Holstein, Ratzeburger Allee 160, D-23538 Luebeck, Germany; Department of Rhythmology, University Heart Center Lübeck, University Clinic Schleswig-Holstein, Ratzeburger Allee 160, D-23538 Luebeck, Germany; Department of Rhythmology, University Heart Center Lübeck, University Clinic Schleswig-Holstein, Ratzeburger Allee 160, D-23538 Luebeck, Germany; Department of Rhythmology, University Heart Center Lübeck, University Clinic Schleswig-Holstein, Ratzeburger Allee 160, D-23538 Luebeck, Germany; Department of Internal Medicine III, University Medical Center of Schleswig-Holstein—Campus Kiel, Kiel, Germany; Department of Electrophysiology and Rhythmology, Herz- und Diabeteszentrum NRW, Ruhr-Universitaet Bochum, Bad Oeynhausen, Germany; Department of Electrophysiology and Rhythmology, Herz- und Diabeteszentrum NRW, Ruhr-Universitaet Bochum, Bad Oeynhausen, Germany; Department of Electrophysiology and Rhythmology, Herz- und Diabeteszentrum NRW, Ruhr-Universitaet Bochum, Bad Oeynhausen, Germany; Department of Internal Medicine III, University Medical Center of Schleswig-Holstein—Campus Kiel, Kiel, Germany; Department of Internal Medicine III, University Medical Center of Schleswig-Holstein—Campus Kiel, Kiel, Germany; Department of Rhythmology, University Heart Center Lübeck, University Clinic Schleswig-Holstein, Ratzeburger Allee 160, D-23538 Luebeck, Germany; Department of Rhythmology, University Heart Center Lübeck, University Clinic Schleswig-Holstein, Ratzeburger Allee 160, D-23538 Luebeck, Germany; German Center for Cardiovascular Research (DZHK), Partner Site Hamburg/Kiel/Lübeck, Luebeck, Germany; Department of Rhythmology, University Heart Center Lübeck, University Clinic Schleswig-Holstein, Ratzeburger Allee 160, D-23538 Luebeck, Germany; Department of Rhythmology, University Heart Center Lübeck, University Clinic Schleswig-Holstein, Ratzeburger Allee 160, D-23538 Luebeck, Germany

**Keywords:** Atrial fibrillation, Cryoballoon, Pulse field ablation, Vascular closure device, Complications

## Abstract

**Aims:**

Simplified ablation technologies for pulmonary vein isolation (PVI) are increasingly performed worldwide. One of the most common complications following PVI are vascular access-related complications. Lately, venous closure systems (VCSs) were introduced into clinical practice, aiming to reduce the time of bed rest, to increase the patients’ comfort, and to reduce vascular access-related complications. The aim of the present study is to compare the safety and efficacy of using a VCS to achieve haemostasis following single-shot PVI to the actual standard of care [figure-of-eight suture and manual compression (MC)].

**Methods and results:**

This is a prospective, multicentre, randomized, controlled, open-label trial performed at three German centres. Patients were randomized 1:1 to undergo haemostasis either by means of VCS (VCS group) or of a figure-of-eight suture and MC (F8 group). The primary efficacy endpoint was the time to ambulation, while the primary safety endpoint was the incidence of major periprocedural adverse events until hospital discharge. A total of 125 patients were randomized. The baseline characteristics were similar between the groups. The VCS group showed a shorter time to ambulation [109.0 (82.0, 160.0) vs. 269.0 (243.8, 340.5) min; *P* < 0.001], shorter time to haemostasis [1 (1, 2) vs. 5 (2, 10) min; *P* < 0.001], and shorter time to discharge eligibility [270 (270, 270) vs. 340 (300, 458) min; *P* < 0.001]. No major vascular access-related complication was reported in either group. A trend towards a lower incidence of minor vascular access-related complications on the day of procedure was observed in the VCS group [7 (11.1%) vs. 15 (24.2%); *P* = 0.063] as compared to the control group.

**Conclusion:**

Following AF ablation, the use of a VCS results in a significantly shorter time to ambulation, time to haemostasis, and time to discharge eligibility. No major vascular access-related complications were identified. The use of MC and a figure-of-eight suture showed a trend towards a higher incidence of minor vascular access-related complications.

## Graphical Abstract

**Graphical Abstract:**
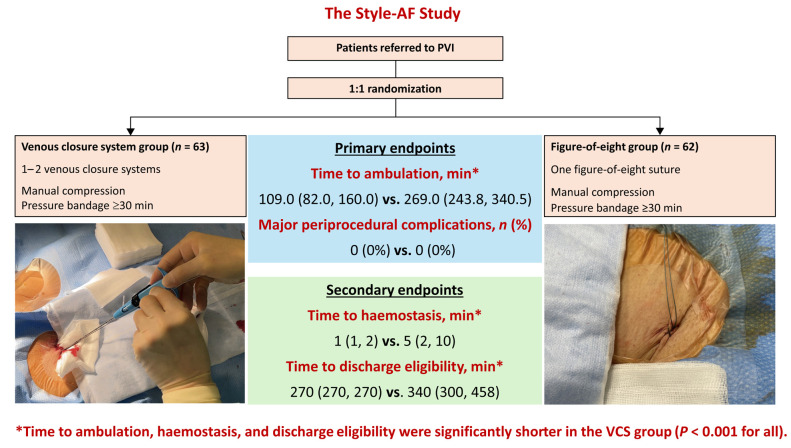
PVI, pulmonary vein isolation.

**What’s new?**

- The use of venous closure systems to achieve vascular haemostasis after electrophysiological procedures was associated with a reduced time to ambulation, time to haemostasis, and time to discharge eligibility.
- No major vascular access-related complications were identified in either group.
- The satisfaction of the patients with the bed rest in the venous closure system group was significantly higher.
- The use of venous closure systems opens the way for the implementation of same-day discharge electrophysiological procedures in clinical practice.

## Introduction

Increasing numbers of patients with indication for pulmonary vein isolation (PVI) as the cornerstone of atrial fibrillation (AF) ablation resulted in the development of widely adoptable, simplified ablation technologies with shorter learning curves and less complication rates, such as cryoballoon-based PVI or pulsed field ablation (PFA).^1–4^

These developments are of particular interest in the light of limited health economic resources with implementation of same-day discharge (SDD) protocols, aiming at improvement of patients’ satisfaction and access to medical services while reducing the costs of medical care.^5–8^ Despite considerable technical advances in the last years, vascular access-related complications remain one of the most frequent complications and the leading cause of delayed discharge following catheter-based AF ablation.^2,9,10^

The incidence of major vascular complications, like pseudoaneurysm, arteriovenous fistula, and retroperitoneal bleeding, is 1.5% and increases with number of sheaths and sheath size.^11^ Furthermore, AF ablation necessitates an intensive periprocedural anticoagulation regime to prevent thromboembolism going along with an increased rate of bleeding and haematoma.^12,13^

Established measures to reduce vascular complications include ultrasound-guided venous puncture, figure-of-eight suture, and manual compression (MC), followed by pressure bandage.^12,14,15^ A short time to ambulation (TTA) and a reduced vascular access complication rate are important prerequisites to successfully implement SDD protocols in real practice. The actual standard of care in achieving haemostasis after venous vascular accesses is MC, with or without a figure-of-eight suture. Several publications have shown that the utilization of a figure-of-eight suture is associated with a shorter time to homeostasis and a lower incidence of bleeding events as compared to the MC alone.^15,16^

The implementation of venous closure systems (VCSs) to achieve haemostasis after electrophysiological procedures has been shown to further reduce the TTA, time to haemostasis (TTH), and time to discharge eligibility (TTDe) while significantly increasing patient satisfaction.^17^

The Perclose™ ProStyle™ and Perclose™ ProGlide™ (Abbott Vascular, CA, USA) vascular closure systems were introduced in clinical practice aiming to address these inconveniences and seem to be a safe and effective alternative to MC for procedures requiring small or large bore vascular accesses.^18,19^

Therefore, the aim of this prospective, randomized study (ClinicalTrials.gov Identifier: NCT05563142) was to compare the efficacy and safety of Perclose™ ProGlide™/ ProStyle™ mediated haemostasis following ‘single-shot’ AF ablation procedures to the actual standard of care: MC and figure-of-eight suture.

## Methods

### Trial design and study participants

The STYLE-AF Study was a prospective, multicentre, randomized, controlled, open-label trial (RCT) performed at three German sites between November 2022 and January 2024. A complete list of inclusion and exclusion criteria as well as their definitions can be found in the Supplementary material S1. Briefly, patients ≥18 years old undergoing elective catheter ablation for AF using a 6–14-Fr inner diameter introducer sheath with a maximum of two femoral venous access sites were enrolled. Exclusion criteria included active systemic or cutaneous infection, or inflammation in the vicinity of the groin, reduced platelet count (<100 000 cells/mm^3^), a body mass index (BMI) > 45 kg/m^2^ or <20 kg/m^2^, attempted or inadvertent arterial puncture, procedural complications that interfered with routine recovery, ambulation, or discharge times, incorrect sheath placement (arterial puncture and placement of the sheath), intraprocedural bleeding or thrombotic complications, or access site-specific eligibility criteria to exclude problems with gaining access or location of sheath.

### Consent

The study protocol, its amendments, the informed consent, and the study worksheets were approved by the Ethics Committee of the University of Lübeck (Reference number: 21-510) and of each individual participating centre. The trial was conducted in accordance with the ethical standards laid down in the 1964 Declaration of Helsinki and its later amendments (ClinicalTrial.gov Identifier https://clinicaltrials.gov/ct2/show/NCT05563142). After fulfilment of all preprocedural inclusion and exclusion criteria, the patients provided the written informed consent at the time of the screening visit.

### Interventions

#### Ablation procedure

The catheter ablation procedures were performed as per institutional standard. The detailed intraprocedural management has been described in previous studies from our centre.^20–24^ Briefly, the interventions were performed under deep sedation. Up to two ultrasound-guided femoral venous punctures were performed for each patient, and only patients with successful venous puncture and without inadvertent or attempted arterial puncture were randomized by means of sealed envelopes. A single transseptal puncture, followed by the insertion of a transseptal sheath, was used to access the left atrium. Heparin boli were administered to target an activating clotting time (ACT) of >300 s. The energy source and procedural details were at the discretion of the centre and operator. The use of protamine at the end of the procedure was allowed as per institutional standard.

##### Study group (venous closure system group)

After successful venous puncture, one VCS [Perclose™ ProGlide™ (Abbott Vascular, CA, USA] or Perclose™ ProStyle™ (Abbott Vascular, CA, USA) was deployed before the insertion of the catheter at a sheath size of ≥9 Fr and another one after the removal of the catheter at a sheath size of <9 Fr. At the end of the procedure, following sheath removal and VCS deployment, MC was applied at the discretion of the operator. A vertical mattress suture (Donati suture) was applied to adapt the cutaneous tissue and to avoid skin bruising (see Supplementary material online, *Video* S*1*). The suture was removed on the next day.

##### Control group [figure-of-eight suture and MC (F8 group)]

A figure-of-eight suture was applied at the femoral venous access site, and the sheaths were removed (Supplementary material online, *Video* S*2*). The haemostasis was achieved exclusively by MC. The time of compression was at the discretion of the operator.

#### Postprocedural management

A pressure bandage was applied for a minimum of 30 min. Serial assessments of the groin were systematically performed by the treating physician (30 min, 60 min, and at least each hour after sheath removal), who decided when the patient was allowed to stand up and walk for 20 ft. The absence of painful swellings requiring drug medication, superficial bleeding, or bruising of the tissue were prerequisites to allow the patient to stand up and walk.

Therapeutic anticoagulation using a vitamin K antagonist (VKA) or a direct oral anticoagulant (DOAC) was resumed per standard of care and continued for at least 3 months postprocedural and thereafter according to the individual CHA_2_DS_2_-VASc score.^1^ Additional medication was administered as per institution’s standard of care and in accordance with current guidelines. The patients discharged on the day of procedure (SDD) were scheduled for an ambulatory visit on the next day postprocedural for the examination of the groin, removal of the suture material, and echocardiographic exclusion of a pericardial effusion.

#### Primary and secondary outcomes

Predefined patient questionnaires assessing comfort/discomfort, pain, and need for pain drugs were performed after the procedure, while 30 days postprocedural, the EQ-5D-5L score was assessed.^12,17,25^ The femoral access site was assessed 30 min after sheath removal, as well as each hour for a minimum of 2 h or until the patient could stand up and walk.

The primary efficacy endpoint was the TTA, defined as the time between the removal of the last sheath and the moment when the patient walked 20 ft without evidence of venous re-bleeding from the femoral access site.

The primary safety endpoint was the incidence of major periprocedural adverse events defined as adverse events until hospital discharge requiring medical intervention. Vascular access complications requiring solely the application of pressure bandage were classified as minor adverse event (see Supplementary materials S2 and S3).

The secondary efficacy endpoints included the TTH, TTDe, and time to discharge (TTD). The complete definitions can be found in the Supplementary material S2.

The secondary safety endpoints included the incidence of major adverse events (adverse events requiring medical intervention or hospitalization) within 30 days after the procedure, incidence of minor adverse events (adverse events not requiring medical intervention or hospitalization) within 30 days after the procedure, procedural success (attainment of final haemostasis at all venous access sites and freedom from major venous access site closure-related complications), and device success (ability to deploy the delivery system, deliver the suture, and achieve haemostasis; see Supplementary material S2).

### Statistical analysis

The study was powered based on the primary efficacy outcome (TTA). Based on the results of the AMBULATE trial, and anticipating a ∼15% increase in the expected TTA in the VCS group but not in the F8 group (pessimist approach) due to use of larger size vascular sheaths (up to 14 Fr vs. up to 12 Fr), the mean TTA for the F8 group was estimated at 6.1 h and for the VCS group at 3.0 h.^17^ With an estimated joint standard deviation of 1.45 h, a sample size of 50 patients per arm would identify the hypothesized difference as significant with a power (1 − *β*) of 100% and a probability of a type I error (*α*) of 0.05 while still retaining sufficient overall numerosity to account for potential outliers. In order to perform a device-based subanalysis (Perclose™ ProStyle™ only), the sample size was subsequently increased to 125 patients (approved by the Ethics Committee of the University of Lübeck—reference number: 2023-495_1, 19.06.2023/CL).

The categorical variables are reported as absolute and relative frequencies [*n* (%)] and were compared using the *χ*^2^ test or the Fisher exact test as appropriate. The continuous variables were tested for normal distribution using the Shapiro–Wilk test. They are reported as mean and standard deviation (mean ± SD) if normally distributed or as median and interquartile range [median (quartile 1, quartile 3)] otherwise. The continuous variables with normal distribution were compared using Student’s *t*-test, while those with not normal distribution using the corresponding nonparametric test (Mann–Whitney *U* test). All *P*-values are two-sided, and a *P*-value of <0.05 was considered statistically significant. All statistical analyses were performed using the SPSS version 29.0 (IBM SPSS Statistics).

## Results

### Study population

**Figure 1:**
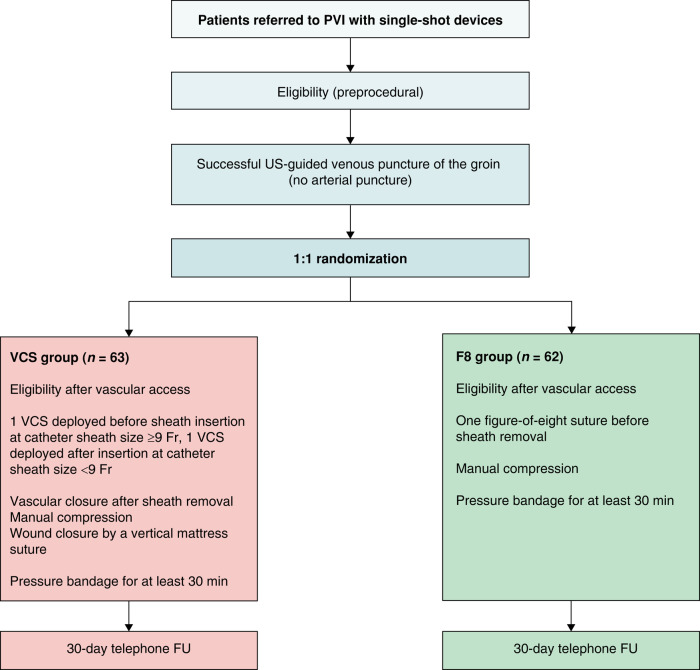
Study flowchart. FU, follow-up; PVI, pulmonary vein isolation.

A total of 125 patients were randomized in a 1:1 fashion in two study arms: 63 patients in the VCS group and 62 patients in the F8 group (*Figure 1*). Excepting the type of AF and BMI, the baseline characteristics of the two groups were similar (*Table 1*). Two patients randomized in the F8 group were treated, due to human error, by means of VCS. The analysis was performed as intention to treat. For two patients in the F8 group, three vascular access sites were implemented. In the VCS, a total of 49 (77.8%) procedures were performed using the Perclose™ ProStyle™ and 14 (22.2%) of them using the Perclose™ ProGlide™ system.

**Table 1:** Baseline characteristic

Variable	VCS group	F8 group	*P*-value
	*N* = 63	*N* = 62	
Female gender, *n* (%)	25 (39.7%)	20 (32.3%)	0.457
Age, years	64.0 (56.0, 74.0)	68.5 (60.8, 75.3)	0.146
Body mass index, kg/m^2^	29.4 ± 5.6	27.5 ± 4.7	0.042*
Arterial hypertension, *n* (%)	40 (63.5%)	36 (58.1%)	0.585
Diabetes mellitus, *n* (%)	5 (7.9%)	7 (11.3%)	0.560
Hyperlipidaemia, *n* (%)	31 (49.2%)	20 (32.3%)	0.069
Smoker, *n* (%)	6 (9.5%)	2 (3.2%)	0.273
Ex-smoker, *n* (%)	5 (7.9%)	4 (6.5%)	1
Chronic kidney disease, *n* (%)	11 (17.5%)	8 (12.9%)	0.619
GFR, mL/min/1.73 m^2^	71.4 ± 18.0	73.8 ± 14.4	0.407
Deep vein thrombosis, *n* (%)	0 (0%)	3 (4.8%)	0.119
Peripheral arterial disease, *n* (%)	1 (1.6%)	1 (1.6%)	1
Coronary artery disease, *n* (%)	15 (23.8%)	15 (24.2%)	1
Cardiomyopathy, *n* (%)	7 (11.1%)	8 (12.9%)	0.789
LVEF, %	55 (54.5, 60.0)	55.0 (52.0, 60.0)	0.829
Sinus rhythm at baseline, *n* (%)	36 (57.1%)	41 (66.1%)	0.359
Atrial fibrillation at baseline, *n* (%)	26 (41.3%)	21 (33.9%)	0.461
Atrial tachycardia at baseline, *n* (%)	1 (1.6%)	0 (0.0%)	1
Atrial fibrillation			
Paroxysmal	32 (50.8%)	44 (71.0%)	0.027*
Persistent	29 (46%)	17 (27.4%)	0.041*
Longstanding persistent	2 (3.2%)	1 (1.6%)	1
Oral anticoagulation, *n* (%)	60 (95.2%)	56 (90.3%)	0.323
DOACs, *n* (%)	59 (93.7%)	52 (83.9%)	0.096
Antiplatelet therapy, *n* (%)	4 (6.3%)	3 (4.8%)	1
CHA_2_DS_2_-VASc			
0	10 (15.9%)	7 (11.3%)	0.603
1	13 (20.6%)	14 (22.6%)	0.830
2	15 (23.8%)	14 (22.6%)	1
3	8 (12.7%)	10 (16.1%)	0.240
4	8 (12.7%)	13 (21.0%)	0.240
5	4 (6.3%)	3 (4.8%)	1
6	5 (7.9%)	1 (1.6%)	0.207
≥3	25 (39.7%)	27 (43.5%)	0.718
HAS-BLED			
0	18 (31.6%)	10 (17.2%)	0.132
1	13 (22.8%)	23 (39.7%)	0.050
2	12 (21.1%)	18 (31%)	0.214
3	12 (21.1%)	5 (8.6%)	0.115
4	1 (1.8%)	2 (3.4%)	0.618
5	1 (1.8%)	0 (0%)	1
≥3	14 (22.2%)	7 (11.3%)	0.150
EHRA score			
I	4 (6.9%)	1 (1.7%)	0.206
IIa	24 (41.4%)	12 (20.3%)	0.016*
IIb	25 (43.1%)	33 (55.9%)	0.197
III	5 (8.6%)	13 (22.0%)	0.071

Values are counts, *n* (%), or mean ± SD or median and interquartile range as appropriate.

DOAC, direct oral anticoagulation; GFR, glomerular filtration rate; LVEF, left ventricular ejection fraction; VCS, venous closure system; *, statistically significant.

### Procedural characteristics

In the VCS group, a total of 51 (81%) procedures were performed using cryoenergy, 11 (17.5%) using PFA, one (1.6%) procedure using laser energy, and no one using RF, while in the F8 group, the distribution was 49 (79.0%), 9 (14.5%), 1 (1.6%), and 3 (4.8%), with no significant difference between the groups (*Table 2*).

**Table 2:** Procedural characteristics

Variable	VCS group	F8 group	*P*-value
	*N* = 63	*N* = 62	
Energy source			
Cryoballoon, *n* (%)	51 (81.0%)	49 (79.0%)	0.826
PFA, *n* (%)	11 (17.5%)	9 (14.5%)	0.808
Laser, *n* (%)	1 (1.6%)	1 (1.6%)	1
RF, *n* (%)	0 (0.0%)	3 (4.8%)	0.119
Number of punctures			
1, *n* (%)	9 (14.3%)	8 (12.9%)	1
2, *n* (%)	54 (85.7%)	52 (83.9%)	0.808
3, *n* (%)	0 (0.0%)	2 (3.2%)	0.243
Ultrasound-guided punctures, *n* (%)	63 (100%)	62 (100%)	
Inadvertent arterial puncture, *n* (%)	0 (0%)	0 (%)	
Procedure duration, min	48.0 (40.0, 59.0)	48.0 (36.5, 59.3)	0.481
Area dose product, cGy·cm^2^	611.0 (396.0, 1200.0)	610 (358.3, 1000.8)	0.485
Fluoroscopy duration, min	8.0 (5.0, 11.0)	8.0 (5.0, 11.0)	0.876
Amount of contrast medium, mL	60.0 (50.0, 80.0)	60.0 (50.0, 70.0)	0.520
Amount of heparin, UI	12 500 ± 3349.3	12 532.3 ± 2785.9	0.953
Protamine use, *n* (%)	3 (4.8%)	6 (9.7%)	0.323
Amount of protamine, UI	7000 (5000, –)	7000 (6500, 7750)	0.440
Perclose™ ProStyle™	49 (77.8%)		
Perclose™ ProGlide™	14 (22.2%)		
VCS failure	2 (3.2%)		

Values are counts, *n* (%), or mean ± SD or median and interquartile range as appropriate.

PFA, pulsed field ablation; RF, radiofrequency; VCS, venous closure system.

In the VCS group, 9 (14.3%) patients received one vascular puncture, 54 (85.7%) two vascular punctures, and no patient three vascular punctures, while in the F8 group, 8 (12.9%) patients received one vascular puncture, 52 (83.9%) two vascular punctures, and 2 (3.2%) three vascular punctures, with no significant difference between the groups. All vascular punctures were ultrasound guided, and no arterial puncture was noted. All vascular accesses were obtained in the right groin.

The two groups were also similar in terms of procedural duration, time and dose of radiation, and amount of contrast medium used. Protamine was administered in 3 (4.8%) patients in the VCS group and in 6 (9.7%) patients in the F8 group (*P* = 0.323). The amount of heparin and protamine administered did not differ between the groups.

### Primary efficacy endpoint

The TTA was significantly shorter in the VCS group as compared to the control group [109.0 (82.0, 160.0) vs. 269.0 (243.8, 340.5); *P* < 0.001] (*Table 3*).

**Table 3:** Times

Variable	VCS group	F8 group	*P*-value
	*N* = 63	*N* = 62	
TTA, min	109.0 (82.0, 160.0)	269.0 (243.8, 340.5)	<0.001*
TTH, min	1 (1, 2)	5 (2, 10)	<0.001*
TTDe, min	270 (270, 270)	340 (300, 458)	<0.001*
TTD, days	2 (2, 3)	2 (2,3)	0.690
Same-day discharge, *n* (%)	9 (14.3%)	7 (11.3%)	0.789

Values are counts, *n* (%), or mean ± SD or median and interquartile range as appropriate.

TTA, time to ambulation; TTH, time to haemostasis; TTD, time to discharge; TTDe, time to discharge eligibility; VCS, venous closure system; *, statistically significant.

### Primary safety endpoint

No major vascular complication, as defined in the study protocol, was reported in either group until discharge. Moreover, no major periprocedural adverse events were noted until discharge in either group.

### Secondary endpoints

The TTH was significantly shorter in the VCS group [1 (1, 2) min] as compared to the F8 group [5 (2, 10) min; *P* < 0.001]. The TTDe was also shorter in the treatment group as compared to the control group [270 (270, 270) vs. 340 (300, 458) min; *P* < 0.001], while the TTD was similar between the groups [2 (2, 3) days for both; *P* = 0.690]. A total of 9 (14.3%) patients in the VCS group and 7 (11.3%) patients in the F8 group were discharged on the day of procedure (SDD; *P* = 0.789).

### Periprocedural non-vascular adverse events

In one case in the VCS group, an intraprocedural aspiration was suspected and the patient was treated with antibiotics. The patient was asymptomatic during follow-up. In 2 (3.2%) patients in the VCS group, the vascular closure system failed to achieve haemostasis and a figure-of-eight suture followed by MC was applied. In one case, a VCS was exchanged due to inability to obtain haemostasis. In this case, three VCSs were used to close two vascular accesses.

Another patient in the VCS group presented a vasovagal syncope with head injury postprocedural. An intracranial haemorrhage was excluded, and the evolution was stable.

One patient (VCS group) with preprocedural bifascicular block and intermittent 2:1 atrioventricular block underwent pacemaker implantation. One patient in the VCS group and two patients in the MC group showed phrenic nerve palsy, which resolved until the end of procedure (*n* = 1) or until discharge (*n* = 2). In patients showing intraprocedural low oesophageal temperature, an upper endoscopy was routinely performed. In one of them (MC group), a mild oesophageal oedema was reported. In one patient in the MC group with postprocedural dysarthria in the wakeup room, a cranial computed tomography was performed to exclude a cerebrovascular event. A neurological event was excluded by a neurologist.

### Vascular access-related complications on the first day postprocedural

The incidence of minor vascular access-related complications was similar between the groups at 30 and 60 min postprocedural, while a trend towards more complications on the day of procedure was noted in the F8 group [15 (24.2%) vs. 7 (11.1%) patients with complications; *P* = 0.063; *Table 4*]. On the day of ablation, significantly more patients in the F8 group exhibited a groin haematoma <6 cm as compared to the VCS group [9 (14.5%) vs. 2 (3.2%); *P* = 0.029]. When analysing the first 2 days postablation, the patients in the F8 group also exhibited a higher incidence of minor vascular access-related complications [22 (35.5%) vs. 13 (20.6%) patients with complications; *P* = 0.075] as compared to those in the VCS group. The incidence of groin pain and the need for pain medication were similar between groups.

**Table 4:** Minor vascular complications and pain

Variable	VCS group	F8 group	*P*-value
	*N* = 63	*N* = 62	
**Complications and pain by 30 min postop**			
Patients with complications, *n* (%)	2 (3.2%)	2 (3.2%)	1
Groin haematoma >6 cm, *n* (%)	1 (1.6%)	0 (0%)	1
Groin haematoma <6 cm, *n* (%)	1 (1.6%)	1 (1.6%)	1
Bleeding, *n* (%)	0 (0%)	1 (1.6%)	0.496
Groin pain, *n* (%)	12 (19%)	14 (22.6%)	0.665
Need for painkillers, *n* (%)	7 (58.3%)	8 (57.1%)	1
**Complications and pain by 1 h postop**			
Patients with complications, *n* (%)	5 (7.9%)	2 (3.2%)	0.440
Groin haematoma >6 cm, *n* (%)	1 (1.6%)	0 (0%)	1
Groin haematoma <6 cm, *n* (%)	2 (3.2%)	2 (3.2%)	1
Bleeding, *n* (%)	2 (3.2%)	0 (0%)	0.496
Groin pain, *n* (%)	10 (15.9%)	8 (12.9%)	0.800
Need for painkiller, *n* (%)	5 (50.0%)	3 (37.5%)	0.664
**Complications present on the day of procedure (total)**			
Patients with complications, *n* (%)	7 (11.1%)	15 (24.2%)	0.063
Groin haematoma >6 cm, *n* (%)	1 (1.6%)	0 (0.0%)	1
Groin haematoma <6 cm, *n* (%)	2 (3.2%)	9 (14.5%)	0.029*
Bleeding, *n* (%)	4 (6.3%)	7 (11.3%)	0.363
Groin pain, *n* (%)	16 (25.4%)	17 (27.4%)	0.841
Need for painkiller, *n* (%)	11 (68.8%)	10 (58.8%)	0.721
**Complications present on the first day postprocedural**			
Patients with complications, *n* (%)	8 (12.7%)	15 (24.2%)	0.111
Groin haematoma >6 cm, *n* (%)	2 (3.2%)	1 (1.6%)	1
Groin haematoma <6 cm, *n* (%)	4 (6.3%)	13 (21.0%)	0.020*
Bleeding, *n* (%)	2 (3.2%)	1 (1.6%)	1
Haemoglobin drop, *n* (%)	0 (0%)	1 (1.6%)	0.496
Groin pain, *n* (%)	8 (12.7%)	10 (16.1%)	0.619
Need for painkiller, *n* (%)	2 (25%)	2 (20%)	1
**Complications present on the day of procedure and the day after that (total)**			
Patients with complications, *n* (%)	13 (20.6%)	22 (35.5%)	0.075
Groin haematoma >6 cm, *n* (%)	2 (3.2%)	1 (1.6%)	1
Groin haematoma <6 cm, *n* (%)	6 (9.5%)	15 (24.2%)	0.033*
Bleeding, *n* (%)	6 (9.5%)	8 (12.9%)	0.584
Haemoglobin drop, *n* (%)	0 (0.0%)	1 (1.6%)	0.496
Groin pain, *n* (%)	16 (25.4%)	21 (33.9%)	0.332
Need for painkiller, *n* (%)	11 (68.8%)	11(52.4%)	0.500

Values are counts, *n* (%), or mean ± SD or median and interquartile range as appropriate.

VCS, venous closure system; *, statistically significant.

### Postprocedural comfort

The median level of satisfaction with the lying time was significantly higher in the VCS group [9.0 (6.0, 10.0)] as compared to the F8 group [7.0 (5.0, 8.0); *P* = 0.016], while a trend towards a higher comfort level was observed in the same group [8.0 (4.0, 9.8) vs. 6.5 (5.0 vs. 8.0); *P* = 0.312]. The severity of the pain was similar between groups. No difference was identified between groups regarding the satisfaction, comfort, and pain level of the current procedure as compared to previous procedures (*Table 5*).

**Table 5:** Satisfaction questionnaires

Question	VCS group	F8 group	*P*-value
**All patients**			
How satisfied are you with the lying time you had to spend on your back?	9.0 (6.0, 10.0)	7.0 (5.0, 8.0)	0.016*
How comfortable was it for you to have to lie on your back?	8.0 (4.0, 9.8)	6.5 (5.0, 8.0)	0.312
How severe was the pain when you had to lie on your back?	1.0 (0.0, 3.0)	1.0 (0.0, 3.0)	0.588
**Venous closure**			
How would you have felt if the postablation time had been 2–3 h longer?	3.0 (2.0, 5.0)		
How comfortable would it have been for you to lie for 2–3 h longer?	3.0 (2.0, 5.0)		
How severe do you think the pain would be if you had to lie on your back for 2–3 h longer?	4.0 (2.0, 7.0)		
**Figure-of-eight**			
How would you have felt if the lying time after ablation had been 2–3 h shorter?		8.0 (6.0, 10.0)	
How comfortable would it have been for you if you had lain for 2–3 h shorter?		8.0 (6.8, 10.0)	
How severe do you think the pain would be if you had to lie on your back for 2–3 h less?		1.0 (0.0, 3.0)	
**Patients with previous procedures**			
How satisfied are you with the lying time compared to previous procedures?	8.0 (5.0, 9.0)	7.5 (5.0, 8.8)	0.251
How comfortable was the lying time compared to previous procedures?	8.0 (5.0, 9.0)	7.0 (5.0, 8.0)	0.546
In your estimation, how severe would the pain be compared to previous procedures?	3.0 (1.0, 5.3)	3.0 (0.0, 5.0)	0.585

Values are median and interquartile range.

VCS, venous closure system; *, statistically significant.

### Thirty days follow-up

No difference was noted between the groups in terms of mobility, fend for oneself, capacity to perform general activities, pain and physical complaints, or anxiety/depression as assessed by the EQ-5D-5L questionnaire at 30 days (*Table 6*).

**Table 6:** EuroQol EQ-5D-5L

Variable	VCS group	F8 group	*P*-value
	*N* = 60	*N* = 57	
Mobility			
0, *n* (%)	50 (83.3%)	45 (78.9%)	0.638
1, *n* (%)	4 (6.7%)	5 (8.8%)	0.739
2, *n* (%)	4 (6.7%)	4 (7.0%)	1
3, *n* (%)	1 (1.7%)	2 (3.5%)	0.612
4, *n* (%)	1 (1.7%)	1 (1.8%)	1

Variable	VCS group	F8 group	*P*-value
	*N* = 60	*N* = 57	
Fend for oneself			
0, *n* (%)	58 (96.7%)	53 (93.0%)	0.4310
1, *n* (%)	1 (1.7%)	2 (3.5%)	0.6122
2, *n* (%)	0 (0.0%)	0 (0.0%)	
3, *n* (%)	0 (0.0%)	1 (1.8%)	0.4872
4, *n* (%)	1 (1.7%)	1 (1.8%)	1

Variable	VCS group	F8 group	*P*-value
	*N* = 60	*N* = 58	
General activities (e.g. work, study, housework, family, or leisure activities)			
0, *n* (%)	46 (76.7%)	44 (75.9%)	1
1, *n* (%)	8 (13.3%)	7 (12.1%)	1
2, *n* (%)	3 (5.0%)	5 (8.6%)	0.4868
3, *n* (%)	2 (3.3%)	1 (1.7%)	1
4, *n* (%)	1 (1.7%)	1 (1.7%)	1

Variable	VCS group	F8 group	*P*-value
	*N* = 59	*N* = 57	
Pain/physical complaints			
0, *n* (%)	47 (79.7%)	45 (78.9%)	1
1, *n* (%)	9 (15.3%)	7 (12.3%)	0.7891
2, *n* (%)	3 (5.1%)	4 (7.0%)	0.7144
3, *n* (%)	0 (0.0%)	1 (1.8%)	0.4914
4, *n* (%)	0 (0.0%)	0 (0.0%)	

Variable	VCS group	F8 group	*P*-value
	*N* = 60	*N* = 57	
Anxiety/depression			
0, *n* (%)	47 (78.3%)	39 (68.4%)	0.2951
1, *n* (%)	10 (16.7%)	10 (17.5%)	1
2, *n* (%)	3 (5.0%)	5 (8.8%)	0.4832
3, *n* (%)	0 (0.0%)	3 (5.3%)	0.1125
4, *n* (%)	0 (0.0%)	0 (0.0%)	
**Current state**	80 (70, 90)	80 (60, 90)	0.378

Values are counts, *n* (%), or median and interquartile range. Scale: level of problems regarding the parameter in five dimensions (0 = low to 4 = high).

VCS, venous closure system.

Regarding postprocedural adverse events, one patient in the VCS group reported a medical visit for the inspection of the haematoma (present at discharge), which did not require any intervention, while another patient in the F8 group had an ambulatory medical visit due to the development of a haematoma. Two patients in the VCS group were readmitted, one because of myocardial infarction with percutaneous coronary intervention and one because of recurrence of AF. One patient in the F8 group was admitted because of fever and suspected pneumonia. No major vascular access-related complications were reported.

### Perclose™ ProStyle™ subanalysis

A subanalysis of the Perclose™ ProStyle™ only showed similar results, with shorter TTA, TH, and TTDe in the device group as compared to the F8 group (see Supplementary material S4).

## Discussion

This is the first prospective, randomized controlled trial to compare the efficacy and safety of Perclose™ ProGlide™/ProStyle™ VCS mediated haemostasis following single-shot PVI with the current standard of care: figure-of-eight suture and MC. The main findings of the study are as follows:

1. The TTA was significantly shorter in the VCS group.
2. The TTH and TTDe were shorter in the study group.
3. No acute major vascular complication until discharge was observed in either group.
4. A trend towards more minor vascular access-related complications on the day of procedure and on the first 2 days was observed in the F8 group.
5. The satisfaction and comfort questionnaires postprocedural showed superior results in the VCS group.

Since the FIRE and ICE and CIRCA-DOSE trials demonstrated the non-inferiority of cryoballoon-based PVI as compared to radiofrequency-based ablation, the former became the cornerstone of interventional AF treatment and opened the way for single-shot ablation technologies.^1,2,26^ The single-shot therapeutic arsenal was amended with the introduction in clinical practice of PFA catheters, showing a similar efficacy and safety profiles, but aiming to reduce complications such as phrenic nerve injury or oesophageal fistula.^3,10,23,27–29^ Recently, a single venous puncture PVI approach was introduced aiming for a further streamlining of the procedure and to the reduction of vascular access complications.^24^

Despite the striking technical innovations and increased operators’ experience, vascular access-related complications remain one of the most common complications following catheter-based PVI.^2,21,30^ The current gold standard in achieving haemostasis after venous access remains the MC with or without a figure-of-eight suture followed by a pressure bandage for several hours.^31^ Several studies comparing the use of a figure-of-eight suture in addition to MC alone found the former superior in terms of TTH, TTA, and bleeding incidence.^15,16,32^ In this study, we compared the use of VCS with figure-of-eight suture and MC—the stronger competitor. The introduction of VCS aims to reduce the rate of vascular access-related complications and the postprocedural bed rest time while increasing the patient’s satisfaction and the access to healthcare resources.^17–19^

In our study, the mean TTA was significantly shorter in the VCS group as compared to the F8 group. These results are in line with other studies reporting on the efficacy of different VCS in achieving haemostasis after catheter-based electrophysiological procedures.^17^ While in the AMBULATE trial, the median TTA in the device group was 2.2 (2.0–11.5) h, and the median TTA in our study was 109.0 (82.0, 160.0) min in the VCS group. The difference can be at least partially explained by the higher number of venous access sites in the former study (three or four punctures), which is expected to associate with a higher TTH, TTA, and higher rate of vascular complications.^17^ A large meta-analysis of RCTs studying the use of vascular closure systems for closure of arterial access also found a significantly shorter TTA in the device group, as well as a shorter TTH and TTD when using the VCS.^33^

In the present study, the TTH in the VCS group was significantly shorter as compared to the control group. In the PRO-PVI study, 41.7% of the patients required additional MC longer than 1 min to achieve haemostasis, with a median TTH of 3 (2–15) min.^12^ In the present study, the use of additional MC in the VCS group was at the discretion of the operators and was implemented in all patients, with a median TTH of 1 (1, 2) min. The individual access site analysis in the AMBULATE trial showed a significantly shorter TTH in the VCS group (6.1 ± 3.7 min) as compared to the F8 group (13.7 ± 6.5 min).^17^ As previously discussed, the difference in the absolute TTH in comparison to our study could be explained by the higher number of venous accesses in this study.

No acute major vascular access-related complication was observed in either arm in our study. Thus, a direct comparison between the two therapeutical approaches could not be performed.

These results are in line with the AMBULATE trial and the PRO-PVI study, which did not report any major vascular complications. Further randomized trials are needed to assess the non-inferiority of VCS as compared to MC and figure-of-eight suture in terms of major vascular complications. However, the lack of such complications in three prospective studies treating a total of 213 patients by means of VCS shows a promising safety profile of this approach.^12,17^

A trend towards more minor vascular complications was observed in the F8 group on the day of the procedure. The difference was mainly driven by a higher rate of groin haematoma <6 cm and bleedings in this group. On the first postprocedural day, a trend towards a higher rate of minor vascular complications was observed in the control group, with a significantly higher rate of groin haematoma <6 cm. No difference was noted between the two groups in terms of groin pain and need of pain medication. It is important to note that the BMI was significantly higher in the VCS group as compared to the control group. Previous studies have shown that a higher BMI is associated with a similar or increased risk of vascular complications as compared to a lower BMI.^34,35^ In the present study, despite the significantly higher BMI, the VCS group showed a trend towards a lower minor vascular access-related complications rate, which represents another argument in favour of the safety of this approach.

Notably, the incidence of minor vascular complications in this study is higher as expected in procedures performed in high-volume centres, by experienced operators and using ultrasound guidance. This aspect reflects the thoroughness of assessing the minor vascular complications, which might be otherwise omitted.

The patients in the VCS group showed a higher level of satisfaction and a trend towards a higher level of comfort regarding the time lying on the back as compared to the F8 group. Moreover, the patients in both groups expressed a higher satisfaction and comfort with the shorter bed rest time when asked to imagine an equivalent change in the bed rest time. Previous studies assessing the satisfaction and comfort of the patients undergoing catheter ablation procedures and VCS-based haemostasis showed similar results, with significantly higher scores in this group as compared to the F8 group.^17^ In contrast to the previously mentioned study, we did not identify a statistically significant difference in terms of pain during bed rest between the groups. Interestingly, with a median score of 9.0 and 8.0 for bed rest time satisfaction and comfort, our results are similar with those reported in the PRO-PVI study (8 and 7.9, respectively), while the median score of 1 for pain by bed rest in the present study is considerably lower as that reported in the previously mentioned study (median 7.8).^12^ Regarding the five dimensions of the EQ-5D-6L questionnaire, no difference was noted between the groups at 30 days follow-up.

### Limitations

The STYLE-AF Study is a multicentre, prospective, randomized, controlled open-label trial; however, there are some limitations. Due to the design of the trial, the study arm could be blinded neither for the patient nor for the physician. Hence, the influence of placebo effect cannot be excluded.

All sites participating in the trial are tertiary electrophysiology centres in Germany, and the procedures were performed by highly experienced operators. Thus, an appropriate learning curve might be necessary to achieve similar results in real practice.

Patients were treated using mostly two venous access sites. Whether the results can be applied for patients with a different number of venous punctures has to be proven.

Same-day discharge was not performed in two out of three participating centres. In one centre, SDD was only offered to highly selected patients. Therefore, the impact of VCD on the SDD rate could not be evaluated in this study.

The assessment of the TTA might be partially subjective. However, the decision of ambulation was based on many predefined criteria in order to increase the objectivity and reduce the inter-physician variability. Moreover, a degree of subjectivity in deciding the ambulation can equally apply for both groups. It is important to note that our approach was rather conservative in the VCS group, where the TTA could have been further reduced, and rather aggressive in the F8 group, where other studies reported TTA up to 6 h after figure-of-eight suture and MC.^36^ A further reduction of this time might be possible in both groups. The TTA is the parameter used in most clinical studies aiming to assess the effectiveness of haemostasis after catheter ablation procedures.^12,17^

The TTDe was reported as per institution’s standard, and different approaches between the study sites were noted.

Two patients randomized in the F8 group were treated by means of VCS due to human error, while two patients randomized in the F8 group were treated using three vascular accesses. However, the analysis was performed per an intention-to-treat basis.

No acute vascular access-related major complication was observed; therefore, no direct comparison was possible. Further studies with a larger population are needed to assess this endpoint.

The procedural use of protamine was not standardized but used as per institutional standard. However, the use of protamine, as well as the amount of protamine used, did not significantly differ between the groups.

## Conclusions

Following PVI, the use of a VCS results in a significantly shorter TTA, TTH, and TTDe. No major vascular access-related complications were identified. More minor complications were reported in the F8 group on the day of procedure. The patients’ satisfaction in the VCS group was significantly higher.

## Supplementary Material

euae105_Supplementary_Data

## Data Availability

Data supporting this study are curated at the Study Centre of the Department of Rhythmology, University Hospital Schleswig-Holstein, Germany. Data are not shared openly but are available on reasonable request from the corresponding authors.
