# Supplementary material for: Venous vascular closure system vs. figure-of-eight suture following atrial fibrillation ablation: the STYLE-AF Study
Source: Europace. 2024 Apr 22;26(5):euae105. doi: 10.1093/europace/euae105 (PMC11210072; doi:10.1093/europace/euae105)
Supplement: euae105_Supplementary_Data [file euae105_Supplementary_Data.zip › Supplementary materials.docx]

1. **Supplementary material S1. Inclusion and exclusion criteria**

**Inclusion criteria**

1. Age ≥ 18 years
2. Elective catheter ablation for atrial fibrillation using a 6 to 14 Fr inner diameter introducer sheath with a minimum of 1 and maximum of 2 femoral venous access sites

**Exclusion criteria**

1. 1.Active systemic or cutaneous infection, or inflammation in vicinity of the groin
2. Platelet count < 100,000 cells/mm3
3. BMI > 45 kg/m2 or < 20 kg/m2
4. Attempted femoral arterial access or inadvertent arterial puncture
5. Procedural complications that interfered with routine recovery, ambulation, or discharge times
6. Incorrect sheath placement
7. Intraprocedural bleeding or thrombotic complications
8. Access site-specific eligibility criteria to exclude problems with gaining access or location of sheath
9. **Supplementary material S2. Primary and secondary endpoints**

Primary efficacy endpoint:

1. Time to ambulation (TTA) after sheath removal. Time to ambulation is defined as the elapsed time between removal of the final closure device or removal of the final sheath and the moment when the patient can stand and walk 20 feet without evidence of venous re-bleeding from the femoral access site.

Primary safety endpoint:

1. Incidence of major periprocedural adverse events defined as adverse events until hospital discharge requiring medical intervention. Vascular access complications requiring solely the application of a pressure bandage will be classified as minor adverse event. (See also Supplementary material S3)

Secondary efficacy endpoints:

1. time to haemostasis (TTH; time elapsed between removal of the closure device or the sheath and first observed and confirmed venous haemostasis, for each access site)
2. time to discharge eligibility (TTDe) (time elapsed between removal of the final closure device or final sheath and when the patient was eligible for hospital discharge based solely on the assessment of the access site, as determined by the medical team)
3. time to discharge (TTD; time elapsed between removal of the final closure device or final sheath and when the patient was discharged from the institution)

Secondary safety endpoints:

1. Incidence of major adverse events within 30 days after the procedure. Major adverse events are defined as complications requiring medical intervention or hospitalisation.
2. Incidence of minor adverse events within 30 days after the procedure. Minor adverse events are defined as AEs not requiring medical intervention.
3. Procedure success (Attainment of final haemostasis at all venous access sites and freedom from major venous access site closure-related complications)
4. Device success (Ability to deploy the delivery system, deliver the suture and achieve haemostasis with vascular closure system (per access site analysis, treatment arm only))
5. **Supplementary materials S3. Definition of access site related adverse events**

**Minor adverse events**

- 1. Access site-related hematoma > 6 cm documented by ultrasound
  2. Localized access site infection confirmed and treated with antibiotics
  3. Arteriovenous fistula not requiring treatment
  4. Pseudoaneurysm requiring thrombin injection or ultrasound guided manual compression
  5. Transient access site-related nerve injury
  6. Vascular access complication requiring application of a new pressure bandage

**Major adverse events**

1. Access site-related bleeding requiring blood transfusion
2. Vascular injury requiring surgical intervention
3. Access site-related sepsis requiring intravenous antibiotics and/or extended hospital stay
4. New onset of persistent access site-related nerve injury (> 30 days)
5. New onset of access site-related nerve injury requiring surgical intervention
6. Pulmonary embolism requiring any intervention and/or resulting in death, confirmed by CT pulmonary angiography or autopsy.
7. **Supplementary materials S4. ProStyle only subanalysis**

| **Variable** | **VCS Group (ProStyle only)**  **N=49** | **F8 Group**  **N=62** | **p-Value** |
| --- | --- | --- | --- |
| TTA, min | 100.0 (80.0, 145.0) | 269.0 (243.8, 340.0) | <0.001 |
| TTH, min | 1 (1, 1.5) | 5 (2, 10) | <0.001 |
| TTDe, min | 270 (270, 270) | 340 (300, 458) | <0.001 |
| TTD, days | 2 (2, 3) | 2 (2,3) | 0.879 |
| Same day discharge, n (%) | 8 (16.3%) | 7 (11.3%) | 0.578 |

Values are counts, n (%) or mean ± SD or median and interquartile range as appropriate.

TA = time to ambulation, TH = time to haemostasias, TTDe = time to discharge eligibility, TTD = time to discharge.
