# Supplementary figures and images for: Venous vascular closure system vs. figure-of-eight suture following atrial fibrillation ablation: the STYLE-AF Study
Source: Europace. 2024 Apr 22;26(5):euae105. doi: 10.1093/europace/euae105 (PMC11210072; doi:10.1093/europace/euae105)

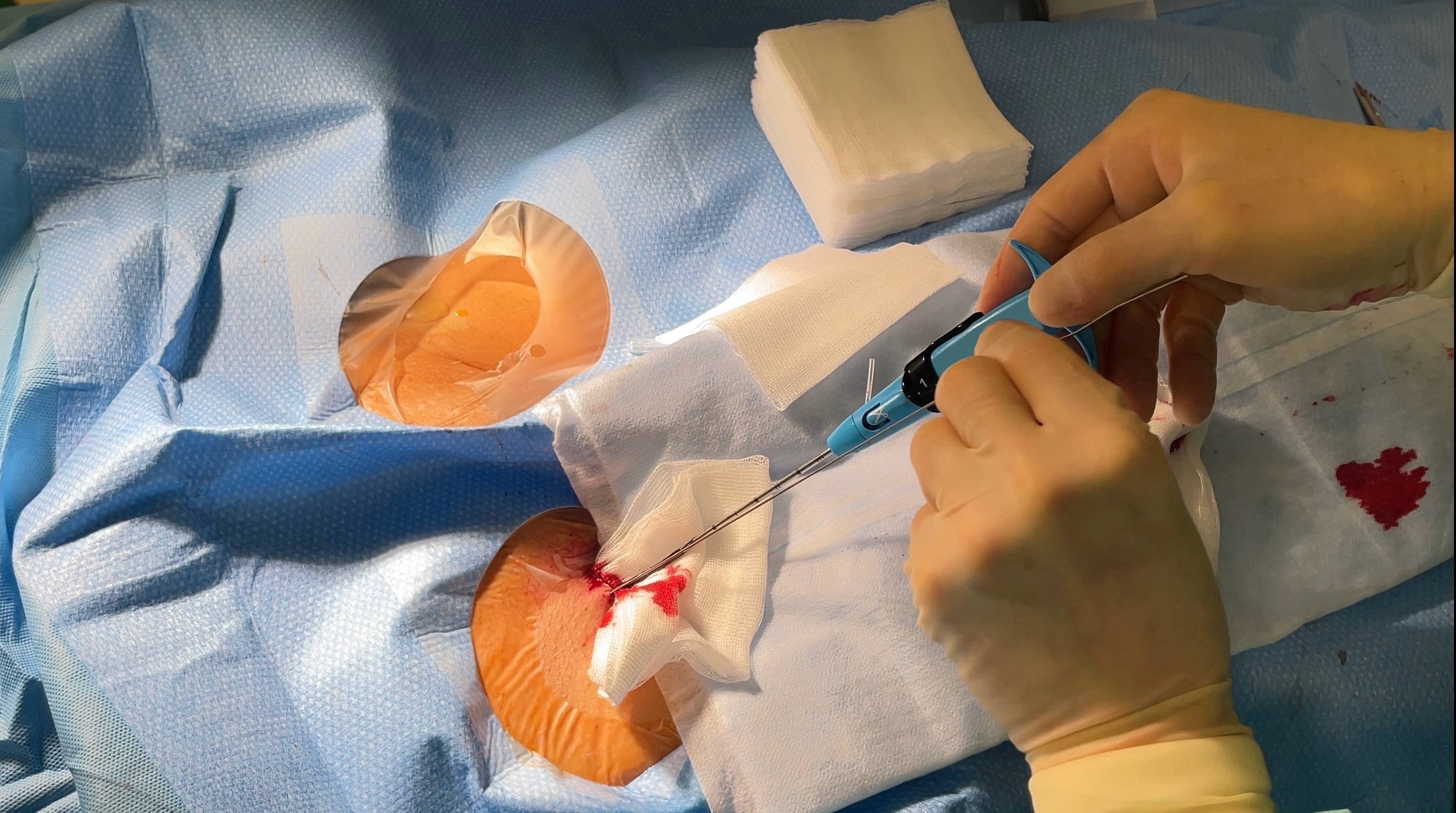

Supplement: euae105_Supplementary_Data [file euae105_Supplementary_Data.zip › Video 1 still image.jpg]

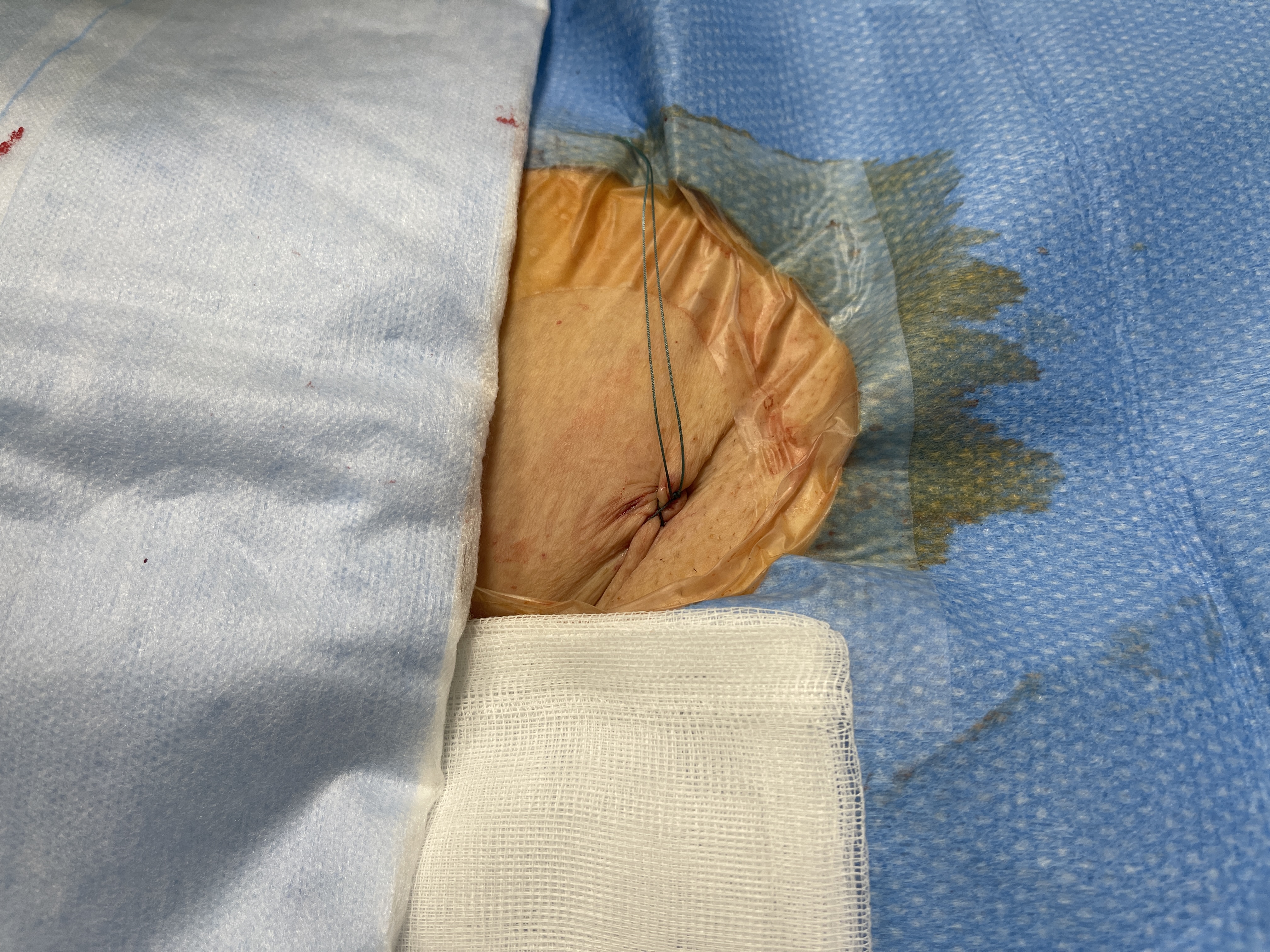

Supplement: euae105_Supplementary_Data [file euae105_Supplementary_Data.zip › Video 2 still image.JPG]
